# The gram-positive HtrA, the protease that is also a chaperone

**DOI:** 10.1128/jb.00360-25

**Published:** 2025-12-23

**Authors:** Sarah Latimer, Charles Agbavor, Laty A. Cahoon

**Affiliations:** 1 Department of Biological Sciences, University of Pittsburgh 171653, Pittsburgh, Pennsylvania, USA; University of Illinois Chicago, Chicago, Illinois, USA

**Keywords:** gram-positive bacteria, HtrA, serine protease, chaperone, secretion

## Abstract

High-temperature requirement A (HtrA) aids in protein homeostasis by playing a key dual role as a chaperone and protease. HtrA ensures protein folding quality control during secretion and protects cells against protein aggregation by degrading misfolded proteins. HtrA proteins are typically composed of a protease domain and at least one PDZ domain, proposed to help regulate their activity and interactions with substrates. In gram-positive bacteria, HtrA contributes to critical cellular functions and has been linked to processes such as maintaining envelope integrity, stress resistance, and virulence. In addition, HtrA has been shown to contribute to the modulation of competence and biofilm dynamics as well as the degradation of host proteins in infection models. In some gram-positive bacteria, HtrA expression is regulated by two-component systems, but many HtrA upstream signals and downstream targets remain unclear. As antibiotic resistance continues to rise, HtrA is gaining attention as a promising target of inhibition for new antibacterial strategies. However, a lack of structural information, unclear regulatory mechanisms, and unknown substrates make designing effective HtrA inhibitors challenging. This review highlights these knowledge gaps and aims to spark more focused research on HtrA in gram-positive species.

## INTRODUCTION

High-temperature requirement A (HtrA) proteins are fascinating bacterial multitaskers that help organisms cope with several types of conditions, such as protein secretion, protein misfolding, oxidative stress, antibiotic resistance, and host infection (1–3). To survive these constant pressures, gram-positive bacteria rely on specialized secretion stress responses. In bacteria, more than one-third of the proteome is secreted across the cell membrane (4). Secreted proteins are translocated by several secretion systems including the Tat and Sec pathways (5). The Tat secretion pathway transports folded proteins (6), while the Sec secretion pathway translocates proteins in an unfolded or loosely folded state (5). In gram-positive bacteria, upon exit from the Sec secretion translocon, protein chains are folded into their native form in the cell wall-cell membrane interface. This space is highly challenging to protein folding because of the dense negative charge generated by the anionic polymers known as teichoic acids, which can be cell wall-attached to the peptidoglycan (wall teichoic acids) or membrane-anchored (lipoteichoic acids) (7). Additionally, the cell wall-cell membrane space is solvent-exposed, which may provide additional challenges for protein folding (7). The mechanism of post-translocation protein folding is critical for bacterial physiology and disease causation, and among the most important responders are chaperones and proteases that help manage protein traffic across the membrane (8). HtrA has been hypothesized to be responsible for protein quality control of translocated proteins at the bacterial cell surface. In gram-positive bacteria, HtrA proteins are predicted to be tethered to the membrane by a transmembrane (TM) domain while the majority of the protein is surface- and solvent-exposed (7). Although the location where HtrA exerts its role is known, there is still much to be discovered regarding its function.

**Fig 1:**
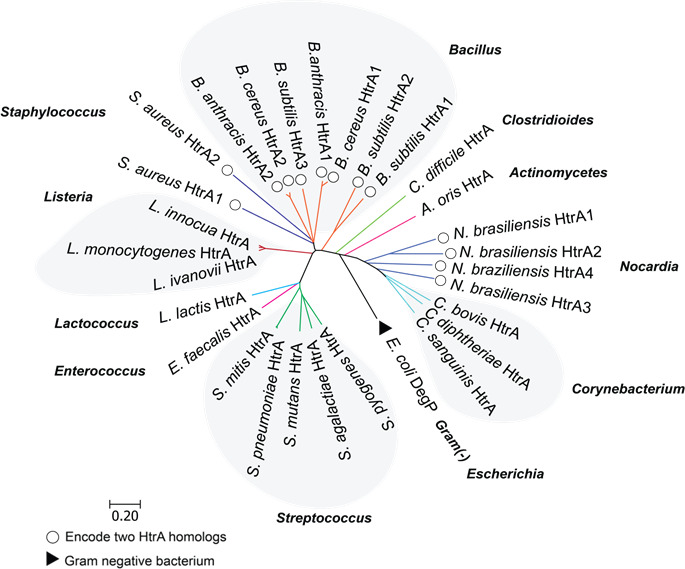
Conservation of HtrA proteins in gram-positive bacteria. A phylogenetic tree of HtrA proteins from representative gram-positive bacterial families is shown. The tree includes 11 genera and 21 bacterial species. A neighbor-joining method with 10,000 bootstraps iterations and Poisson model was used to create the tree in MEGA v11.0.13 (14). Bacterial species with more than one HtrA protein (circles) and a gram-negative bacterium (triangle) is indicated. The scale bar indicates 1 substitution for every 20 amino acids (AA).

HtrA proteins are conserved across all domains of life and have been defined as proteases but have also been shown to act as ATP-independent protein chaperones that assist in protein folding (9). This conservation suggests critical roles in protein quality control and adaptation to cellular stress (2). Further, HtrA proteins exhibit a remarkable degree of domain conservation across various gram-positive bacterial families, suggesting their importance in bacterial homeostatic processes (10). Unlike their gram-negative counterparts like *Escherichia coli*, which typically have three paralogs of HtrA (DegP, DegQ, and DegS), gram-positive bacteria predominantly have a single HtrA protein with the exception of bacillus species, *Staphylococcus aureus*, and *Nocardia braziliensis,* which harbor two or more HtrA homologs (11, 12) (Fig. 1). HtrA is interesting because of its dual identity: as a chaperone to fold proteins or as a protease to degrade proteins (9). In gram-negative bacteria, research suggests that the decision between protease or chaperone may be based on cues from the environment or through changes regulated by the PDZ domain (13). Despite the reported importance of HtrA in stress adaptation in bacteria, the molecular mechanisms surrounding its activity, specifically in gram-positive bacteria, remain poorly understood as compared to their gram-negative counterparts.

Multiple gram-positive species such as *Listeria monocytogenes*, *Streptococcus pneumoniae*, *S. aureus*, *Bacillus subtilis*, and *Bacillus anthracis* rely on HtrA for various responses such as resistance to temperature, osmotic stress, and oxidative stress (3, 11, 12, 15–17). In addition to these stress-responsive roles, gram-positive HtrA proteins are implicated in virulence and processes such as invading host tissues and resistance to antibiotics (2). An example is *S. pneumoniae*, where deletion of HtrA impairs lung infection and reduces bacterial adhesion to respiratory tract cells (3). Similarly, *L. monocytogenes* HtrA cleaves host extracellular matrix (ECM) proteins, promoting bacterial dissemination and infection (18). Since HtrA has critical functions in both bacterial survival and virulence, the protein has gained attention as a target for therapeutic development (9, 19). Inhibitors of HtrA may offer a new approach to treat bacterial infections, particularly in the ever-growing fight against antibiotic resistance. Recent studies have explored the development of small-molecule inhibitors targeting the proteolytic activities of HtrA (9, 19). Although this work is still in its early stages and notably studied in gram-negative bacteria, these efforts offer promising potential for the development of therapeutic strategies targeting bacterial virulence and stress adaptation to halt or minimize host invasion. Here, we will review the reported biological functions of HtrA in gram-positive bacteria while emphasizing the need to urgently address knowledge gaps. We hope to motivate new research focused on expanding the characterization of HtrA functions and mechanisms and hence tackle bacterial infections and challenges posed by antibiotic resistance more effectively. This review will explore known functions of HtrA in gram-positive bacterial survival and virulence by discussing HtrA structure, conservation, regulatory mechanisms, and the potential of future inhibitors.

## STRUCTURAL AND FUNCTIONAL CHARACTERISTICS OF HTRA PROTEINS IN GRAM-POSITIVE BACTERIA

**Fig 2:**
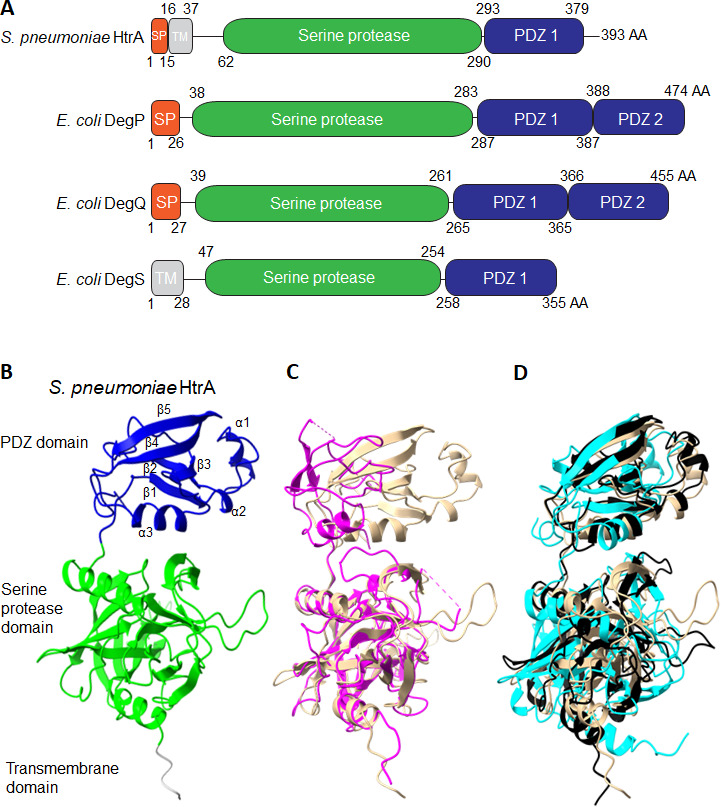
Domain organization and structure of HtrA proteins in gram-positive bacteria. (**A**) Comparison of domain organization of the HtrA protein in bacteria. A representative gram-positive HtrA protein from *S. pneumoniae* is compared to the three HtrA proteins from *E. coli* drawn to scale. The SP, TM domain, serine protease domain (green), and PDZ domain(s) (blue) are indicated where numbers represent AA. (**B**) *S. pneumoniae* HtrA- predicted structure using Alphafold2 (26, 27). The TM domain (gray), serine protease domain (green), and PDZ domain (blue) are indicated. The three α-helices and five β-strands of the PDZ domain are labeled. (**C**) Multiple structure alignment of the Alphafold-predicted structure of *S. pneumoniae* HtrA (cantaloupe, AF-A0A4J1YLW5) and the solved structure of *E. coli* DegS (pink, PDB: 3STI, chain A) (27, 28). *S. pneumoniae* HtrA aligns to *E. coli* DegS with a root mean square deviation (RMSD) of 0.88 Å across all carbon atoms. (**D**) Multiple structure alignment using Alphafold-predicted structures of HtrA proteins from diverse gram-positive bacteria including *S. pneumoniae* (cantaloupe; AF-A0A4J1YLW5), *L. monocytogenes* (black; AF-A0A7L8R4W6), and *B. cereus htrA1* (cyan; AF-B7I048) (27, 28). Structures align well to *S. pneumoniae* HtrA with the RMSD < 3.0 Å.

HtrA proteins are found in all domains of life (10). In gram-positive bacteria, HtrA proteins are conserved in all bacteria families (Fig. 1). HtrA proteins typically contain an N-terminal serine protease domain linked to at least one C-terminal PDZ domain (named after the first three PDZ domain-containing proteins identified, 95 kDa post-synaptic density protein, the *Drosophila melanogaster* disks-large protein, and the zonula occludens 1 protein) (Fig. 2A) (20–23). Additionally, gram-positive bacterial HtrA proteins often contain a signal peptide (SP) at the N-terminus that directs the protein chain for secretion through the Sec translocon and a TM domain that spans the bacterial cell membrane (Fig. 2A and B). The serine protease domain contains a chymotrypsin-like protein fold and a Ser/His/Asp catalytic triad (an oxyanion hole and substrate-specificity pockets) that is essential for the degradation of damaged or misfolded protein chains at the cell membrane-cell wall interface (24, 25). Gram-positive HtrA proteins typically contain one PDZ domain and are structurally unique from gram-negative DegP and DegQ proteins with two PDZ domains, but similar to DegS, which has one PDZ domain and a TM domain (Fig. 2).

The gram-positive HtrA PDZ domain contains a canonical set of three α-helices and five β-strands that form a β-sandwich structure predicted to regulate the proteolytic activity of the HtrA protein (29) (Fig. 2B). PDZ domains of several protein families maintain their specificity for target substrates even in heterologous systems by directly recognizing the C-terminus of their biological partners (23, 30). Interestingly, PDZ domains present substrates to their linked domains, as observed for the *E. coli* periplasmic tail-specific protease (Tsp) protein (31). So far, at least 20 structures of PDZ domains are solved bound to short peptide chains (32, 33). These data show a common peptide-binding groove, wherein the peptide substrates form an additional β-strand in the peptide pocket bound by the β1-strand and the α3-helix, but the orientations and positions of the AA residues vary, suggesting a mechanism of binding specificity (29, 33). The HtrA peptide pocket formed by the β1-strand and the α3-helix is also reminiscent of the common carboxylate binding loop (G-L-G-F loop) in the PDZ domain of synaptic protein PSD-95 as well as other eukaryotic PDZ domain-containing proteins (34). Because most of the PDZ domain-bound complexes are eukaryotic in origin, there may be differences in the recognitions of peptide substrates between prokaryotic and eukaryotic PDZ domain-containing proteins. Interestingly, the *S. pneumoniae* HtrA PDZ domain was tested for interaction with the peptide KRVYYF, a short C-terminal motif found in *E. coli* misfolded outer membrane proteins (35), and *S. pneumoniae* HtrA PDZ domain-interacting residues were found within the α3-helix and β1-strand (29), suggesting a conserved binding groove in bacteria. Since the PDZ domain is critical for interaction with protein substrates (21), this suggests that the PDZ domain is likely important for modulating the chaperone and protease activities of the HtrA protein.

Compared to prokaryotes, PDZ domain-containing proteins in eukaryotes are usually found in multidomain scaffolding proteins for the assembly of large complexes (36). Indeed, it is important to note that PDZ domains occur very frequently in the human proteome, probably owing to high levels of signaling and/or regulation in multicellular organisms (23). Many HtrA proteins and interacting substrates have been characterized in eukaryotes and gram-negatives such as *E. coli* (37–39). However, the complete HtrA protein structure and substrate specificities are yet to be determined in gram-positive bacteria.

## HTRA, THE PROTEASE WITH CHAPERONE ACTIVITY

Gram-negative HtrA orthologs such as *E. coli* DegS, DegP, and DegQ proteins are often modulated by high stress caused during cellular and/or environmental changes. For example, under high stress conditions such as the accumulation of periplasmic misfolded outer membrane proteins, the *E. coli* DegS PDZ domain binds to the C-terminus of these misfolded proteins, which positions the protease domain catalytic triad in an appropriate conformation to cleave the anti-sigma factor RseA that leads to the activation of sigma factor E (σE) that mediates the envelope stress response (40, 41). In the absence of misfolded proteins, DegS proteolytic activity is maintained inactive by coordination between the serine protease domain and the PDZ domain (42). DegS is thought to function primarily as a protease (43), whereas DegP has the ability to function as either a protease or chaperone (44). The first HtrA ortholog identified was *E. coli* DegP critical for proteolytic activity and growth at high temperatures (45, 46), while at low temperatures, DegP functions as a chaperone (44). Later, a structural basis for DegP chaperone and protease activity was identified where DegP forms up to 24-mer oligomeric structures by binding to misfolded proteins at high temperature (47), and in the proteolytic state, the PDZ domain presents substrates to the serine protease domain for degradation (43). Lastly, *E. coli* DegQ with the same domain architecture as DegP is predicted to function similarly (48). However, DegQ possesses higher chaperone activity and a much lower protease activity compared to DegP (48).

Like their gram-negative counterparts, gram-positive HtrA proteins such as those of *B. subtilis*, *B. anthracis, L. monocytogenes,* and others are critical at elevated temperatures (12, 17, 49, 50) (Table 1). However, whether they directly or indirectly activate stress-induced transcription factors or sigma factors is not clear. The *B. subtilis* YkdA (HtrA or HtrA1) and YvtA (HtrB or HtrA2) may be activated by a sigma-type heat-shock-inducible factor (12, 49). In addition, the mechanism that allows gram-positive HtrA proteins to function as either a chaperone or protease at the atomic level is not known. In addition, there are no solved structures of the gram-positive HtrA protease domain. Overall, further research is needed to establish the mechanism of how gram-positive HtrA switches between chaperone and protease.

**TABLE 1:** Effects of HtrA mutation in physiology and virulence of gram-positive species

Bacterium	Physiology	Virulence	Source(s)
*Bacillus anthracis*	HtrA1 deletion increases sensitivity to heat, ethanol, and oxidative and osmotic stress. HtrA1 deletion induces sporulation induction. HtrA1 PDZ deletion restores stress resistance, but an HtrA1 protease-inactive mutant remains sensitive. HtrA2 is involved in spore germination.	An *htrA1* deletion is highly attenuated for virulence in mice. The HtrA1 protease domain is sufficient for virulence, while a protease-inactive mutant is attenuated. An *htrA1* deletion in strain Vollum shows decreased virulence in the guinea pig model of anthrax.	(17, 51–53)
*Bacillus subtilis*	Inactivation of both HtrA1 (HtrA and YkdA) and HtrA2 (HtrB and YvtA) causes growth defects and thermosensitivity. Protease-inactive HtrA enhances bacterial fitness and enzyme yields. HtrA3 (HtrC and YycK) is involved in spore germination.	Not applicable	(49, 53, 54)
*Clostridioides difficile*	HtrA deletion increases acid sensitivity and reduces spore formation. The HtrA PDZ domain is not required for proteolysis or acid resistance.	An HtrA deletion mutant showed enhanced virulence in the Golden Syrian hamster model of acute infection but showed reduced adherence to colonic cells.	(55, 56)
*Enterococcus faecalis*	HtrA is critical for sortase-assembled pili processing.	HtrA deletion is defective for wound persistence in mice.	(57)
*Lactococcus lactis*	HtrA deletion increases high temperature, ethanol, puromycin, and osmotic stress sensitivity.	Not applicable	(58, 59)
*Listeria monocytogenes*	HtrA deletion increases osmotic, puromycin, high temperature, and oxidative stress sensitivity. HtrA PDZ deletion exhibits reduced proteolytic activity.	HtrA deletion impairs survival in mice. HtrA degrades human ECM proteins (fibrinogen, fibronectin, plasminogen, and casein). HtrA PDZ deletion mutants cleave fewer substrates and have altered ECM-binding affinities.	(15, 18, 60, 61)
*Staphylococcus* *aureus*	In strain COL, both HtrA1 and HtrA2 are critical for thermal stress survival.	In strain RN6390 (but not COL), an *htrA1 htrA2* double-deletion mutant is diminished for virulence in a rat model of endocarditis. HtrA1 mutations in the protease and PDZ domains are common in vancomycin-tolerant isolates.	(11, 62)
*Streptococcus agalactiae*	HtrA deletion alters protein secretion levels.	In mice, an *htrA* deletion increased dissemination to placentas and fetuses but caused significantly fewer adverse pregnancy outcomes compared to wild-type. Recombinant HtrA cleaves human fibronectin *in vitro*.	(63)
*Streptococcus mitis*	HtrA regulates competence.	Unknown	(64)
*Streptococcus* *mutans*	HtrA mutants demonstrate reduced tolerance to low and high temperature, acidic pH, and oxidative stress. HtrA mutants display reduced transformation efficiency and altered colony morphology.	HtrA mutants display altered biofilm formation.	(65–67)
*Streptococcus pneumoniae*	HtrA deletion alters protein secretion levels, increases sensitivity to lysozyme and osmotic stress, and shortens bacterial chains. HtrA regulates competence. High temperature triggers an HtrA-dependent competence pathway.	HtrA deletion impairs invasive lung infection in mice. In cefotaxime-resistant strains, HtrA deletion restores penicillin-binding proteins and increases β-lactam resistance. HtrA is required for dispersal from biofilm at febrile-range temperatures.	(1, 3, 16, 68–72)
*Streptococcus pyogenes*	Unknown	HtrA is critical for the maturation of the virulence factor SpeB.	(73)

## THE DIVERSE GRAM-POSITIVE *HTRA* GENETIC LOCUS

**Fig 3:**
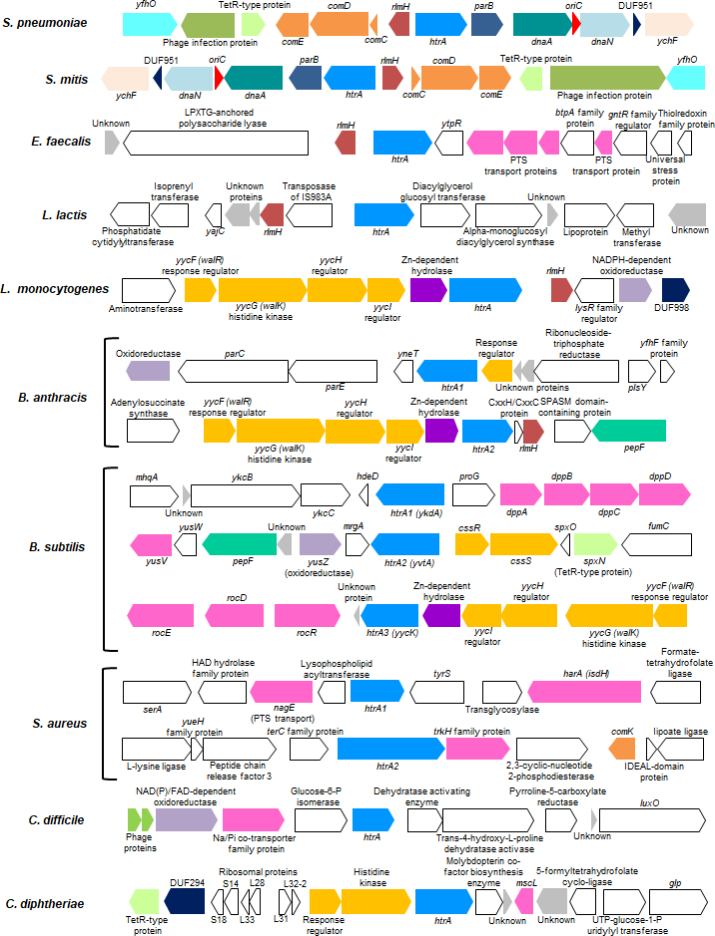
The *htrA* gene locus is diverse across gram-positive bacteria. The locus organization of *htrA* with ~12,000 base pairs surrounding sequence (~6,000 base pairs each side) shown in the 5′ to 3′ orientation from eight genera of gram-positive bacteria. The *htrA* gene locus (blue) and adjacent features are indicated. Open reading frames were predicted using Bakta (82) and rendered using Proksee (83).

The organization of the genes surrounding the *htrA* locus in gram-positive bacteria is highly diverse across families (Fig. 3). However, within the genus Streptococci, *htrA* is commonly located near the origin of replication, *oriC*, and the organization surrounding *htrA* in *S. pneumoniae* and *Streptococcus mitis* appears to be conserved (Fig. 3). In *S. pneumoniae*, *htrA* is transcribed in an operon with *parB (68*), which promotes segregation of replicated chromosomes, and followed by *dnaA*, which initiates the unwinding of DNA during replication together with other protein complexes at *oriC (74*). Interestingly, in *S. pneumoniae* and *S. mitis*, located approximately 2,000 base pairs upstream of *htrA* is the operon *comCDE* encoding the two-component system ComDE, a major regulator of natural competence in addition to the competence-stimulating factor, ComC (75–77). Notably, *L. monocytogenes*, *B. subtilis*, *B. anthracis*, and *Corynebacterium diphtheriae* also appear to encode two-component systems upstream of an *htrA* ortholog (78–80) (Fig. 3). While the *B. subtilis htrA3* has two upstream two-component systems, *yycHI* and *yycFG*, where YycHI regulates YycFG, this organization is also observed upstream of *L. monocytogenes htrA* and *B. anthracis and B. cereus htrA2* (78–80) (Fig. 3). *S. pneumoniae*, *S. mitis*, *B. subtilis*, and *C. diphtheriae* also encode an additional putative TetR transcriptional regulator with a helix-turn-helix motif upstream of *htrA* (Fig. 3). Surprisingly, in *S. aureus*, similar to *S. pneumoniae* and *S. mitis*, a competence transcription factor, in this case, *comK,* is approximately 2,000 base pairs downstream of *htrA2* and is required for the uptake of DNA from the environment but under conditions where respiration of the bacterium is inhibited (81) (Fig. 3). Interestingly, *S. aureus htrA2* is nearly twice the size of *htrA1* and appears to have an additional 5′ end domain predicted to be involved in adhesion to host cells. Notably, bacillus species encode multiple *htrA* homologs where *B. anthracis* and *B. cereus* have similar loci organization; however, only the *B. subtilis htrA3* locus is similar to the *htrA2* locus of *B. anthracis* and *B. cereus*.

Several *htrA* orthologs including *S. pneumoniae*, *S. mitis*, *Enterococcus faecalis*, *Lactococcus lactis*, *L. monocytogenes*, *B. subtilis htrA3,* and *B. anthracis htrA2* are adjacent, either upstream or downstream of the ribosomal large-subunit ethyltransferase H, encoded by *rlmH* (Fig. 3). In *E. coli*, RlmH has been characterized as a 70S ribosome methyltransferase (84), where deletion of *rlmH* produces a growth defect (85). In addition, in *E. coli*, overexpression of RlmH confers a fitness advantage in the presence of ethanol, suggesting RlmH may enhance survival under general stress conditions (86); however, whether RlmH enhances survival in gram-positive bacteria remains to be determined. Interestingly, evidence in *L. lactis* suggests RlmH ribosome modification may act as a defense mechanism to limit group II intron retro-transposition (87); whether this mechanism is conserved in other gram-positive bacteria is unknown.

Further similarities between some of the gram-positive *htrA* orthologs are the presence of genes that encode transporter proteins or components (Fig. 3). *E. faecalis* and *S. aureus htrA1* are adjacent to phosphoenolpyruvate: sugar phosphotransferase system (PTS) components. The PTS is a sophisticated multicomponent system that functions to import several types of carbohydrates, having a central role in metabolism, allowing for bacterial growth on specific substrates, and regulating several cellular processes (88, 89). In *B. subtilis*, *htrA1* is adjacent to *dppABCD*, a dipeptide ABC transport operon that is expressed early during sporulation and may aid in the adaptation to nutrient deficiency (90, 91), while downstream of *htrA2* is *yusV*, an Fe^+3^ siderophore transporter ATP-binding protein that may function as part of two different ABC uptake systems (92). Further, *B. subtilis htrA3* is located adjacent to *rocR* and the *rocDEF* operon involved in arginine import and catabolism (93). In *S. aureus*, downstream of *htrA1* is *harA*, critical for heme binding and uptake (94, 95), while immediately downstream of *htrA2* is a *trkH* family member with a putative role in potassium uptake (96). In *Clostridioides difficile*, upstream of *htrA* is a putative sodium/inorganic phosphate co-transporter family protein, and interestingly, in *C. diphtheriae* downstream of *htrA* is *mscL,* a putative large conductance mechanosensitive channel protein (97). However, although several types of transporter proteins or components are in the vicinity of *htrA*, it is unclear whether HtrA is important directly or indirectly for the function of these proteins. In addition, overall, with the exception of the streptococcal species, the organization of the genes surrounding the *htrA* loci shows few conserved genes in gram-positive bacteria.

## THE MULTIFACETED ROLE OF HTRA IN BACTERIAL PHYSIOLOGY AND VIRULENCE

In several gram-positive bacterial pathogens, HtrA proteins have roles in both physiology and virulence (Table 1). These highly conserved serine proteases with chaperone activity are critical for maintaining protein quality control under normal physiological conditions and especially under stress conditions. By degrading or refolding misfolded proteins, HtrA prevents cellular damage and maintains protein homeostasis (1, 2, 9, 19). Most of this knowledge has been extracted from studies in gram-negative bacteria, and most commonly *E. coli*. For example, *E. coli* DegP was shown to function as a chaperone at cooler temperatures below 28°C and transitions into a protease at hotter temperatures above this threshold (44). The ability to quickly adapt allows gram-negative bacteria to react to changing conditions and aids in fitness and survival. Similar roles have been identified in some gram-positive bacteria, although information is more limited than their gram-negative counterparts.

A role for HtrA in stress tolerance is apparent for several gram-positive bacteria (Table 1). For example, in *S. pneumoniae*, deletion of *htrA* results in increased susceptibility to lysozyme, osmotic stress, and oxidative stress compared to wild-type strains (3, 16). In *C. difficile*, HtrA is essential for acid resistance, with its expression induced at low pH (55). In *L. monocytogenes*, HtrA exhibits high temperature-dependent proteolytic activity, with the PDZ domain enhancing substrate cleavage (18). *B. subtilis* utilizes HtrA1 (YkdA) for stress response regulation during fermentation, where its absence leads to increased secretion stress and lower enzyme yields (54). In *B. anthracis*, HtrA1 is critical for stress tolerance, particularly against oxidative stress and high salt conditions, with its deletion causing widespread transcriptional changes, including downregulation of stress-related regulators and upregulation of sporulation genes as a survival response (51, 52). The role of HtrA in stress tolerance is shared by several additional gram-positive bacteria including *L. lactis*, *S. aureus*, and *Streptococcus mutans* (11, 58, 59, 65, 66) (Table 1).

In streptococcal species including *S. pneumoniae*, *S. mitis*, and *S. mutans*, HtrA is critical for natural competence (3, 16, 64, 67); whether this is a conserved feature of streptococcal species has yet to be determined. HtrA also appears to have roles in cellular morphology such as in *S. pneumoniae* where deletion mutants exhibit significantly shorter bacterial chains, while *S. mutans htrA* mutants produce altered colony morphology and biofilm formation (3, 65). Interestingly, in *S. pneumoniae*, HtrA is required for biofilm dispersal at febrile-range temperatures (69). In addition, HtrA orthologs may have species-specific roles in physiology, for example, *Streptococcus thermophilus* relies on HtrA for surface protein proteolysis such as the cleavage of PrtS, a cell envelope proteinase that enables the bacterium to grow in specific environments (98). Another example is *E. faecalis*, where HtrA functions as a quality control factor for membrane-bound pili, with its deletion causing a higher percentage of aberrant pili-expressing cells (57).

In addition to roles in physiological processes, HtrA orthologs also have critical functions in bacterial virulence and pathogenicity (Table 1). HtrA contributes to bacterial virulence by facilitating host tissue invasion and immune evasion. In *S. pneumoniae,* deletion of HtrA results in impaired invasive lung infection in mice and reduced adhesion to upper respiratory tract cells (1, 3, 16). Although HtrA mutants show decreased colonization in *S. pneumoniae*, the bacterium still manages to establish an infection (3), suggesting there may be additional compensatory networks involving other proteases or chaperones that need further exploration. In *L. monocytogenes*, deletion of *htrA* impairs bacterial survival in mice, and HtrA is proposed to enhance virulence by cleaving host ECM proteins such as fibrinogen, fibronectin, and plasminogen, promoting bacterial dissemination into host cell environments (18, 60, 61). Similarly, recombinant *Streptococcus agalactiae* HtrA was found to directly cleave human fibronectin *in vitro* (63). However, it is unclear how HtrA from either *L. monocytogenes* or *S. agalactiae* is released into the extracellular milieu. In *B. anthracis, htrA1* deletion mutants are also highly attenuated for virulence in mice. Interestingly, the PDZ domain is not required for virulence, indicating that the protease domain is sufficient for infection (52). Another example is *E. faecalis*, where HtrA is implicated in long-term wound colonization, with its deletion resulting in defective persistence in mouse wound models (57) (Table 1). Collectively, HtrA serves several functions in both bacterial survival and virulence, with roles varying across different species and environmental conditions. As HtrA aids in stress resistance and protein homeostasis under physiological conditions, the protein also facilitates colonization and virulence in animal models of infection. However, further research is necessary to elucidate specific HtrA substrates, additional deletion mutant phenotypes, and regulatory mechanisms in gram-positive bacteria.

## REGULATORY DYNAMICS BETWEEN HTRA AND TWO-COMPONENT SYSTEMS

The regulation of *htrA* by two-component systems is critical for stress adaptation for several bacteria. In *S. pneumoniae*, the CiaRH two-component system plays a central role in directly regulating *htrA* expression levels (70, 99–105). CiaR, the response regulator of this system, binds to a conserved promoter element upstream of *htrA*, activating its transcription under stress conditions such as elevated temperature and oxidative stress (100, 103, 105). Hyper-activation of CiaRH causes increased HtrA levels, which in cefotaxime-resistant strains results in the degradation of altered PBP 2×, demonstrating the role of HtrA in modulating β-lactam resistance (71). In *S. pneumoniae*, deletion of either the *ciaRH* two-component system or *htrA* reduces virulence in mouse infection models and increases sensitivity to oxidative stress (100). These findings highlight that HtrA is a key mediator of the virulence effects regulated by the CiaRH system in *S. pneumoniae* (100). CiaR has also been implicated in the regulation of *htrA* in other streptococcal species including *S. mutans* and *Streptococcus sanguinis* (106, 107).

In other gram-positive bacteria, *htrA* is also regulated by two-component systems. In *B. subtilis*, the CssRS two-component regulatory system directly upstream of *htrA2* responds to misfolded proteins at the membrane-cell wall interface by upregulating the expressions of *htrA1* and *htrA2* (79, 108) (Fig. 3). Under secretion or heat stress, CssRS upregulates *htrA1* and *htrA2,* ensuring secreted protein quality control (79, 108, 109). In addition, CssRS induces the expression of the *htrA* promoter in the presence of several cell wall- and membrane-targeting substances, suggesting a general cell envelope stress response (110, 111). In *E. faecalis*, RNA expression analysis suggests *htrA* is part of the vast CroRS two-component system regulon that responds to cell envelope stress (112, 113). In addition, interestingly, the absence of *htrA* activates the CroRS system as a response to an abundance of mislocalized pili on the cell membrane (57). In *E. faecalis*, HtrA normally clears these aberrant pili to maintain envelope integrity, but when HtrA is absent, accumulation of these mislocalized pili triggers the CroRS stress response (57). These findings highlight a direct link between HtrA protein quality control on the cell surface (57). In *L. monocytogenes,* HtrA is downstream of the two-component system *yycFG*; however, it is unclear whether this system regulates the expression of *htrA* as attempts to disrupt the regulator *yycF* have proven unsuccessful, suggesting, as in *B. subtilis*, this regulator is essential (114, 115). In *L. monocytogenes*, evidence suggests *htrA* is regulated by at least two systems including LisRK and PieRS (116, 117); both two-component systems are critical in response to envelope stress and some aspects of virulence (78). Overall, this work underscores the varied regulatory dynamics of *htrA* by multiple two-component system pathways contributing to bacterial adaptation and survival across gram-positive bacteria.

## POTETIAL HTRA INHIBITORS FOR THERAPEUTIC DEVELOPMENT AGAINST GRAM-POSITIVE PATHOGENS

Since HtrA is critical for stress tolerance and virulence in several pathogenic bacteria, developing efficient HtrA inhibitors could revolutionize the control of gram-positive host infections. The function of HtrA in degrading host proteins, enhancing adhesion, and regulating antibiotic resistance underscores its potential as a therapeutic target. The increasing bacterial resistance to antibiotics presents the need to develop new antibacterial strategies. Thus far, research has focused on inhibiting HtrA in gram-negative bacteria, with less focus on gram-positive pathogens. In gram-negative bacteria, several compounds have been identified that interfere with HtrA activity (Table 2). One of the most studied HtrA inhibitors is JO146, a peptide-based compound that irreversibly inhibits *Chlamydia trachomatis* HtrA by forming a covalent bond with its catalytic serine residue (118–122). As a result, the enzyme remains suppressed over time and has been shown to significantly reduce the bacterial viability of *C. trachomatis, Chlamydia muridarum,* and *Chlamydia pneumoniae* in koalas (120, 122). Despite JO146 effectiveness, the peptide composition of this inhibitor makes it unstable and hinders the ability of JO146 to cross membranes, which limits its therapeutic potential (118, 119). JO146 derivatives have been developed with enhanced stability and pharmacokinetic properties that retain inhibitory effects on HtrA (118, 119, 122); however, these compounds remain highly specific for chlamydia species, limiting their application on other bacteria (120). Another class of inhibitors based on a pyrazolo[1,5-a]−1,3,5-triazine structure target *E. coli* DegS (HtrA) and disrupts the PDZ domain (123). This inhibits the activation of the σE stress response pathway, effectively decreasing bacterial survival under stress (123). In addition, these inhibitors have been combined with the antibiotic colistin, which produced stronger antibacterial effects (123). However, because these compounds do not directly block HtrA proteolytic activity but instead interfere with HtrA-dependent stress pathway activation, they may be less effective in bacterial populations that are not exposed to stress-inducing conditions.

**TABLE 2:** Strengths and limitations of key HtrA inhibitory compounds

Compound	Bacterial target	Mode of inhibition	Strengths	Limitations	Source(s)
JO*146*	*Chlamydia trachomatis, Chlamydia muridarum, and Chlamydia pneumoniae*	Irreversible covalent HtrA inhibitor	– Potent selective inhibition – Minimal host off target activity – Impairs bacterial development – Retains efficacy under stress conditions – Effective in human and koala chlamydia *in vivo* models, with no observed cytotoxicity to host cells	– Specific to *Chlamydia* genus – Long treatments may be needed for full efficacy – Current formulation is not suitable for clinical application	(118–122)
pyrazolo[1,5-a]−1,3,5-triazine scaffold compounds	*Escherichia coli*	Allosteric binding to HtrA PDZ domain	– Inhibits DegS with measurable activity – Exhibits synergy with the antibiotic colistin	– High micromolar range is needed for inhibition. – Solubility at high concentrations may pose challenges	(123)
*H. pylori* HtrA inhibitor (HHI)	*Helicobacter pylori*	HtrA protease active site small-molecule inhibitor	– Inhibits HtrA protease activity, preventing E-cadherin cleavage and intercellular spread – Protects host epithelial cells by preserving cellular junction integrity during infection – Targets bacterial HtrA without inhibiting human HtrA1	– Testing is limited to cell models – Demonstrates moderate potency	(124, 125)
Nafamostat mesylate	*Listeria monocytogenes*	Serine protease inhibitor	– Demonstrated efficacy against SARS-CoV-2, supporting antiviral and antibacterial applications – High inhibitory potency against bacterial HtrAh	– Specific mode of inhibition is undefined	(126)
Epigallocatechin gallate (EGCG)	*Listeria monocytogenes*	Serine protease inhibitor, the galloyl group binds S2 pocket and hydroxyl groups interact with Asp 259 and 326	– High binding affinity to *L. monocytogenes* HtrA – Naturally occurring compound with low inherent toxicity	– High concentrations are required for complete inhibition	(127)

HtrA inhibitors have also been explored in *Helicobacter pylori*, where HtrA protease activity functions in pathogenesis by cleaving host E-cadherin (124, 128). Small molecules like HHI and other *de novo* designed small-molecule inhibitors have been shown to prevent E-cadherin cleavage by binding to the HtrA protease active site and limiting the ability of the bacteria to damage gastric intestinal cells (124, 125). Unlike traditional antibiotics which kill bacteria, these inhibitors block a specific virulence mechanism without directly affecting bacterial survival, which may be advantageous because there is less selective pressure to develop bacterial resistance. However, since these inhibitors do not directly kill bacteria, they will likely need to be used in combination with other therapies or traditional antibiotics. Moreover, other gram-negative HtrA inhibitors include metals such as zinc and copper, which are naturally present both in the environment and as essential trace elements within the human body. Zinc and copper inhibit HtrA activity in *H. pylori*, while zinc has been shown to inhibit HtrA activity in *Borrelia burgdorferi* (129, 130). Zinc has been proposed to bind to serine and/or histidine residues in the active site of these bacterial HtrA proteases, thereby preventing substrate access and reducing enzymatic activity, while copper may destabilize the HtrA protease structure or cause oxidative damage (129). In addition, zinc may interact with an HtrA allosteric ligand-binding loop important for oligomer stability identified in *H. pylori* (130). While these metals present an interesting avenue for inhibition, their potential unknown toxicity and impact on host cells pose challenges for current therapeutic use.

In contrast to the advances made in gram-negative bacteria, efforts to develop HtrA inhibitors for gram-positive pathogens are still underexplored (Table 2). Some serine protease inhibitors, such as camostat mesylate, gabexate mesylate, and nafamostat mesylate, have been tested against *L. monocytogenes* HtrA (126). These compounds interact with the HtrA serine protease active site, effectively blocking its function, and nafamostat mesylate appears to be the most potent of these inhibitors (126). However, these compounds need further optimization because they demonstrate weak binding affinities and/or limited ability to penetrate membranes, which reduces their effectiveness. Natural compounds have also been explored for their potential to inhibit *L. monocytogenes* HtrA. EGCG, a polyphenol found in green tea, has been shown to bind to *L. monocytogenes* HtrA, likely altering its activity through an allosteric mechanism (127). While EGCG is abundant in nature and also consumed widely, to be effective against bacteria, high concentrations are required. In addition, EGCG is unstable and may have off-target effects. Although some progress has been made, significant gaps remain in the search for effective HtrA inhibitors, especially in regard to gram-positive bacteria. Many existing inhibitors suffer from limitations related to specificity, stability, binding affinity, and cell permeability, requiring further optimization. Future research should prioritize structure-based drug design to improve potency and selectivity, as well as explore alternative strategies, such as the modulation of allosteric sites. HtrA-targeting therapeutics could offer valuable new treatment options, especially in the fight against antibiotic-resistant infections.

## PERSPECTIVES: OUTSTANDING GAPS IN HTRA RESEARCH ACROSS GRAM-POSITIVE BACTERIA

HtrA is involved in several important bacterial processes from protein quality control to stress response, virulence regulation, and biofilm dynamics. However, major gaps remain in our mechanistic understanding of how this protease and chaperone functions, how it is regulated, and why HtrA is critical for bacterial physiology and pathogenesis. In *S. pneumoniae*, for instance, HtrA is critical for bacterial competence, acting in parallel with csRNAs under the control of the CiaRH two-component system (104); while HtrA is important for competence in other streptococcal species, it is unclear whether the mechanisms observed in *S. pneumoniae* are more widely applicable to these other organisms. Also, the specific substrates through which HtrA has influence on the ComDE signaling system are unknown, and although ComD and the competence-stimulating peptide were ruled out as direct targets, the identity of the HtrA-dependent effector remains a mystery (68). Moreover, how the activity of HtrA is coordinated with other regulators like Spx in *B. subtilis*, which is involved in oxidative stress responses (131), or other pathways tied to the heat shock response, in addition to regulation by two-component systems, has yet to be explored in depth. These interactions could help clarify the role of HtrA in competence, which may tie into other stress-related cellular processes and regulatory networks.

Another recurring gap in knowledge across nearly all studied gram-positive species is detailed HtrA substrate specificity. Whether HtrA acts as a general chaperone and/or protease interacting with all secreted proteins is unclear, although proteomic evidence in *S. pneumoniae* suggests that HtrA may have a specific substrate repertoire (3). In addition, although shown in gram-negative bacteria, HtrA chaperone activity has not been definitively demonstrated in gram-positive bacteria. Further, there is limited information regarding the gram-positive HtrA structure, and it is unknown whether the enzyme forms the characteristic multimers observed in their gram-negative counterparts (55). In *L. monocytogenes*, HtrA has been shown to cleave host ECM components such as fibrinogen and plasminogen (18); however, there is a lack of structural data to explain the mechanism of these interactions. This limitation is exacerbated by the absence of a solved gram-positive HtrA crystal structure, which also limits the potential for inhibitor development.

Despite these gaps in our knowledge, luckily, although limited, HtrA has drawn some attention as a therapeutic target (Table 2). HtrA has been implicated in the virulence and stress survival of several gram-positive pathogens (Table 1), making the protein a strong candidate for inhibitor development. Finally, there is a pressing need for broader comparisons across species to further understand general HtrA mechanisms versus species-specific mechanisms. These gaps emphasize the need for the use of integrative approaches combining transcriptomics, proteomics, and structural biology to explore the full extent of HtrA function across gram-positive bacteria. While much progress has been made, the field of HtrA research in gram-positive bacteria still holds a plethora of research opportunities. Addressing the mechanistic, regulatory, and functional gaps outlined here and beyond will be critical for unlocking the therapeutic potential of this intriguing protease and chaperone while fully understanding its contribution to bacterial processes.
